# Transient receptor potential vanilloid four in macrophages mediates TGF-β activation to drive myofibroblast differentiation and pulmonary fibrosis

**DOI:** 10.1016/j.jbc.2026.111135

**Published:** 2026-01-07

**Authors:** Lisa M. Grove, Caitlin Snyder, Adam M. Boulton, Hongxia Mao, Susamma Abraham, Haley Ricci, Erica M. Orsini, Brian D. Southern, Mitchell A. Olman, Rachel G. Scheraga

**Affiliations:** 1 Inflammation and Immunity, Cleveland Clinic Research, Cleveland Clinic, Cleveland, Ohio, USA; 2 Integrated Hospital Care Institute, Department of Pulmonary and Critical Care, Cleveland Clinic, Cleveland, Ohio, USA

**Keywords:** lung macrophages, TGF-b activation, pulmonary fibrosis, cytoskeleton, actin, myosin

## Abstract

Emerging evidence suggests that macrophage-fibroblast interactions can drive organ fibrosis. Myofibroblast differentiation is a key step in the pathogenesis of pulmonary fibrosis that requires both a soluble (*e.g.*, TGF-β) and mechanical signal. We have previously implicated the fibroblast mechanosensitive cation channel, transient receptor potential vanilloid 4 (TRPV4), as a mediator of myofibroblast differentiation and experimental pulmonary fibrogenesis in response to matrix biophysical signals. Less is understood regarding how or if the matrix drives macrophage activation to mediate fibrosis. We demonstrate that loss of TRPV4 specifically in myeloid cells protects against experimental pulmonary fibrosis *in vivo*. Mechanistically, macrophage TRPV4 responds to matrix substrate stiffness in the pathophysiologic range, thereby optimizing TGF-β activation. Macrophage conditioned media transfer and coculture systems demonstrate a profound effect of TRPV4-dependent TGF-β activation in inducing myofibroblast differentiation in fibroblasts. This TGF-β activating effect was dependent on the actinomyosin binding domain within the C-terminal intracytoplasmic tail of TRPV4 and on assembly of actinomyosin cytoskeleton and its force generation. Our current study identifies a novel TRPV4-TGF-β axis in macrophages that drives myofibroblast differentiation and experimental pulmonary fibrosis through optimal activation of TGF-β. As TGF-β is a critical pro-fibrotic factor, these findings are broadly applicable to many fibrotic diseases.

Fibrotic interstitial lung diseases (fILDs) are devastating progressive disorders that carry a high mortality and lack a cure (1, 2). The hallmark of fILDs is accumulation of pathogenic fibroblasts referred to as myofibroblasts (3). Myofibroblasts have distinct pro-fibrotic characteristics including changes in motility, secretion of extracellular matrix proteins (*e.g.*, collagen, fibronectin, and matrix components) and pro-fibrotic cytokines (*e.g.*, TGF-β) (4). Conditions where myofibroblast development, pro-fibrotic actions, and clearance are not tightly regulated in a spatio-temporal manner leads to progressive fibrosis (5, 6). Understanding the key signals that mediate myofibroblast differentiation are essential for ameliorating fibrosis in many organs.

The myofibroblast differentiation response to extracellular matrix mechanical force is essential to the development of fibrosis. Emerging evidence suggests mechanosensitive cation channels such as Piezo 1, Piezo 2, and transient receptor potential vanilloid 4 (TRPV4) are important in the pathogenesis of pulmonary fibrosis (7, 8, 9, 10). Our group has specifically implicated TRPV4 in fibroblasts in TGF-β induced fibroblast to myofibroblast transdifferentiation and experimental pulmonary fibrosis (8, 9). We additionally have shown that macrophage activation also depends on matrix stiffness, an effect not recognized previously, as *in vitro* experiments were performed on supraphysiologic tissue culture plastic and glass, which have stiffness a million times that of lung tissue (11, 12). While ubiquitously expressed, TRPV4 has been increasingly recognized to have cell-type and context-specific actions, likely as a consequence of TRPV4 crosstalk with other signaling pathways (13). This crosstalk could be mediated through TRPV4’s intracytoplasmic amino- and carboxy-terminal tails or its cation channel function (14, 15).

Fibroblasts have long been identified as a key effector cell in fibrosis, whereas the contribution of immune cells has been more controversial (16, 17, 18, 19). More recently it has been shown that alveolar macrophages persist at the leading edge of fibrosis, the fibroblastic foci, and evidence supports their important contribution to fibrogenesis, albeit through an unclear mechanism (17, 20, 21). Thus, this work was initiated to uncover previously unknown macrophage TRPV4-dependent, pro-fibrotic actions. Here, we identify TRPV4 in macrophages as a key driver of TGF-β activation that induces myofibroblast differentiation and mediates experimental pulmonary fibrosis. We found that optimal matrix substrate stiffness-dependent TGF-β activation in macrophages requires actinomyosin-induced force, and the presence of the actinomyosin binding site within TRPV4’s C-terminal intracytoplasmic tail. This novel macrophage TRPV4-TGF-β axis may function as a druggable target to ameliorate organ fibrosis.

## Results

### TRPV4 deletion in myeloid cells protects the lung from bleomycin-induced pulmonary fibrosis

**Figure 1:**
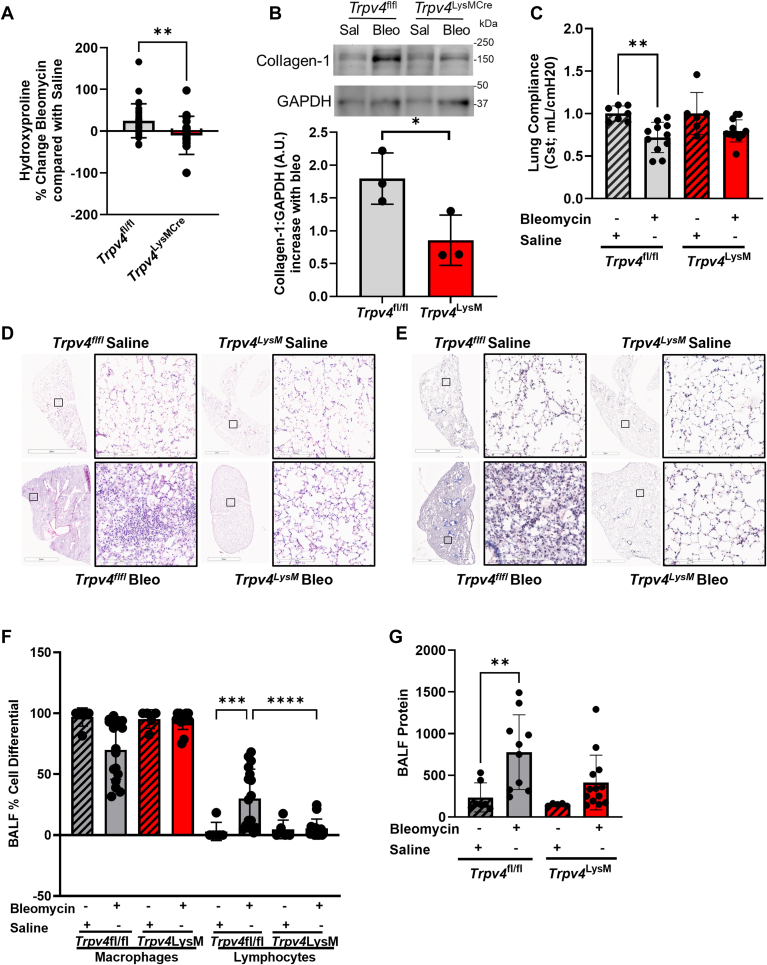
**TRPV4 deletion from myeloid cells protects the lung from pulmonary fibrosis.***Trpv4*^fl/fl^ and *Trpv4*^LysMCre^ mice were intratracheally instilled with saline (*hashed bars*) or 1.5 U/kg bleomycin (*solid bars*) and all analysis at Day 14. *A* hydroxyproline content increased in the lungs after bleomycin of *Trpv4*^fl/fl^ as compared to *Trpv4*^LysMCre^ mice. Results shown as mean ± SD for four independent experiments with 22 to 28 mice/group (shown as individual points). ∗∗*p* < 0.01 *Trpv4*^fl/fl^*versus Trpv4*^LysMCre^ mice (unpaired, 2-tailed *t* test). *B*, collagen-1:GAPDH was measured in pooled whole lung homogenate from saline and bleomycin treated *Trpv4*^fl/fl^ and *Trpv4*^LysMCre^ mice. Results shown as mean ± SD for three independent experiments (shown as individual points) with 22 to 28 mice/group pooled lysate. ∗*p* < 0.05 *Trpv4*^fl/fl^*versus Trpv4*^LysMCre^ mice (unpaired, 2-tailed *t* test). *C,* lung Compliance (Cst; mL/cmH20) was measured using FlexiVent. Results shown as mean ± SD for four independent experiments with 6 to 11 mice/group (shown as individual points). ∗∗*p* < 0.01 ± bleomycin *Trpv4*^fl/fl^ (ANOVA/Holm-Sidak’s multiple comparisons test). *D,* representative photomicrographs of H&E-stained lung tissue from saline and bleomycin treated *Trpv4*^fl/fl^ and *Trpv4*^LysMCre^ mice. The scale bar represents 2 mm (*zoomed out*) and 200 μm (*zoomed in*). *E,* representative photomicrographs of Trichrome-stained lung tissue from saline and bleomycin treated *Trpv4*^fl/fl^ and *Trpv4*^LysMCre^ mice. This scale bar represents 2 mm (*zoomed out*) and 200 μm (*zoomed in*). *F,* bronchoalveolar lavage fluid cell differentials were measured from both genotypes with increased lymphocytes in *Trpv4*^fl/fl^ mice treated with bleomycin. Results shown as mean ± SD for four independent experiments with 7 to 16 mice/group (shown as individual points). ∗∗∗*p* < 0.001 ± bleomycin *Trpv4*^fl/fl^, ∗∗∗∗*p* < 0.0001 *Trpv4*^fl/fl^*versus Trpv4*^LysMCre^(ANOVA/Fisher’s LSD). *G,* total protein increased in bronchoalveolar lavage fluid in *Trpv4*^fl/fl^ as compared to *Trpv4*^LysMCre^ mice. Results shown as mean ± SD for four independent experiments with 5 to 13 mice/group (shown as individual points). ∗∗*p* < 0.01 ± bleomycin *Trpv4*^fl/fl^(ANOVA/Fisher’s LSD).

To investigate the role of TRPV4 in myeloid cells on *in vivo* pulmonary fibrogenesis, the effect of 1.5 U/kg intratracheal instillation of bleomycin or saline (Day 14) was studied in myeloid specific *Trpv4* KO mice (*Trpv4*^LysMCre^), as compared with parental controls (*Trpv4*^fl/fl^). The myeloid specific *Trpv4* KO mice (*Trpv4*^LysMCre^) were significantly protected from bleomycin-induced fibrosis (n >/ = 5–20 mice per group). This was assessed by the complementary techniques of lung hydroxyproline content by >50% (Fig. 1*A*), collagen-1 levels in lung tissue by immunoblotting (Fig. 1*B*), lung compliance (Fig. 1*C*), and representative flow-volume loop in Fig. S1. Lung histologic analysis of H&E (Fig. 1*D*) and trichrome stained (Fig. 1*E*) lung tissue revealed more prominent fibrosis in parental controls compared to the myeloid specific *Trpv4* KO (*Trpv4*^LysMCre^). To assess the extent of lung injury, bronchoalveolar lavage analysis revealed decreased lymphocyte infiltration (by 25%) (Fig. 1*F*) without a change in total cell counts (data not shown), and decreased bronchoalveolar lavage fluid total protein (by 50%) (Fig. 1*G*) in the myeloid specific *Trpv4* KO (*Trpv4*^LysMCre^). These data collectively support the conclusion that TRPV4 in the myeloid population mediate fibrogenesis in response to bleomycin *in vivo*.

### Macrophages produce a TRPV4 and matrix stiffness-dependent factor that induces myofibroblast differentiation

**Figure 2:**
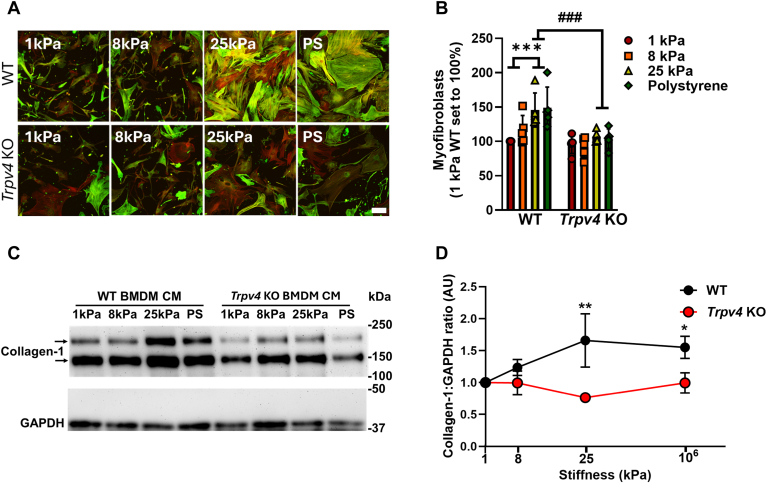
**TRPV4 in macrophages is required for secretion of a stiffness-dependent, pro-fibrotic factor that induces myofibroblast differentiation.** Bone marrow derived macrophages (BMDMs) from WT and *Trpv4* KO mice were differentiated with M-CSF for 7 days, plated on pathophysiologic range matrix stiffnesses (1 kPa: normal lung, *red bar*; 8 kPa: fibrotic lung, *orange bar*; 25 kPa: fibrotic lung, *yellow bar*; Polystyrene (10^6^ kPa) standard culture conditions, *green bar*), and conditioned media (CM) was transferred to WT mouse lung fibroblasts (MLFs). *A.* CM from WT BMDMs induced myofibroblasts in MLFs in a stiffness-dependent manner, an effect lost upon deletion of TRPV4 in macrophages as measured by immunofluorescence and quantified in *B.* results shown as mean ± SD from five independent experiments (shown in individual data points) performed in technical duplicates. ∗∗∗*p* < 0.001 WT 1 kPa and 8 kPa *versus* WT 25 kPa and polystyrene; ^###^*p* < 0.001 WT 25 kPa and polystyrene *versus* KO 25 kPa and polystyrene (ANOVA/Student-Newman-Keuls). The scale bar represents100 μm, 10× original mag. *Green* = alpha smooth muscle actin, *red* = phalloidin (F-actin), *yellow* = merged. *C,* CM from WT BMDMs induced collagen-1:GAPDH production in MLFs in a stiffness-dependent manner, an effect lost upon deletion of TRPV4 in macrophages as measured by immunoblot and quantified in *D,* results shown as mean ± SEM from four independent experiments. WT 1 kPa and *Trpv4* KO 1 kPa each set to 1. ∗∗*p* < 0.01 or ∗*p* < 0.05 WT *versus Trpv4* KO on higher stiffnesses (ANOVA/Fisher’s LSD). BMDM, bone marrow derived macrophage; CM, conditioned media; MLF, mouse lung fibroblast.

Since we previously published that TRPV4 can control macrophage cytokine production in response to toll-like receptor stimulation (11, 12), we now hypothesize that TRPV4 may also play a role in pro-fibrotic macrophage actions. To that end, the effect of the conditioned media from WT bone marrow derived macrophages (BMDMs) and *Trpv4* KO BMDMs plated on polyacrylamide gels of varying stiffness on myofibroblast differentiation was compared. The substrate stiffness’s was chosen in order to recapitulate that of normal (1 kPa) and fibrotic (8, 25 kPa) lung, with supraphysiological tissue-culture plastic (10^6^ kPa) as a positive control. The BMDM conditioned media (CM) (24 h) was then transferred to WT mouse lung fibroblasts (MLF) which was assessed for their myofibroblast differentiation. The CM from WT BMDMs plated on 25 kPa gels and tissue-culture plastic (but not those on 1 kPa and 8 kPa gels) induced myofibroblast differentiation, as measured by staining and quantifying alpha-smooth muscle actin in stress fibers (Figs. 2, *A* and *B* and S6*A*), and by immunobloting for collagen-1 in myofibroblast lysates (Fig. 2, *C* and *D*). As evidence of the importance of TRPV4 in pro-fibrotic macrophage actions, the CM from *Trpv4* KO BMDMs did not induce myofibroblast differentiation, even with supraphysiologic substrate stiffness (Fig. 2, *A*–*D*). These data indicate that a TRPV4 and matrix-stiffness dependent, soluble factor is produced by macrophages that drive myofibroblast differentiation.

### TGF-β is the profibrotic factor in conditioned media that induces myofibroblast differentiation

**Figure 3:**
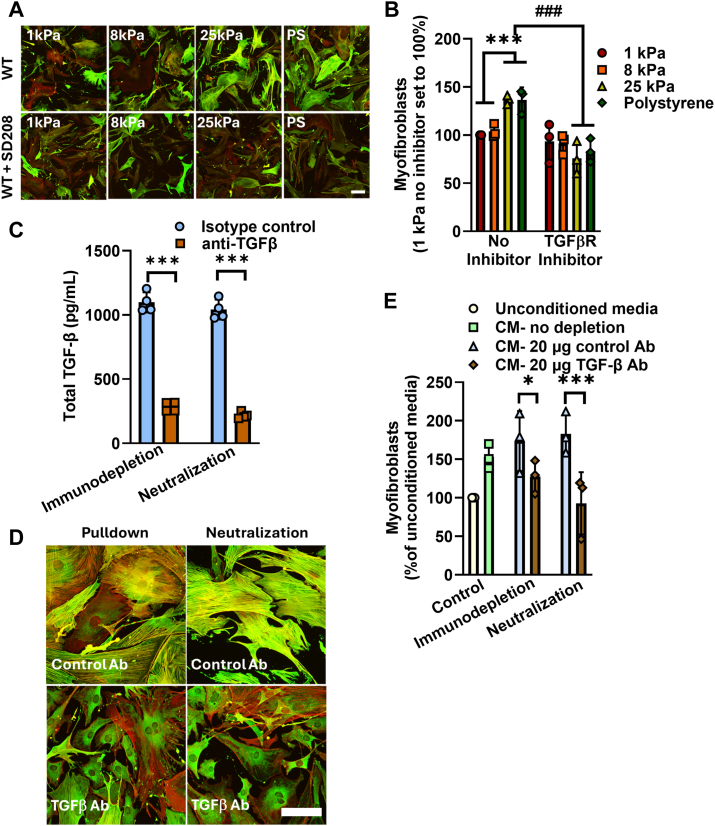
**TGF-β is the matrix stiffness-dependent pro-fibrotic factor secreted by macrophages.** CM from differentiated WT BMDMs that were plated on pathophysiologic range matrix stiffnesses was transferred to WT MLFs ± TGF-β receptor (ALK5) inhibitor (SD208). *A*, SD208 blocked WT BMDM CM’s ability to induce myofibroblasts in a stiffness-dependent manner as assessed by immunofluorescence and quantified in *B,* results shown as mean ± SD from three independent experiments (shown in individual data points) performed in technical duplicates. ∗∗∗*p* < 0.001 WT 1 kPa and 8 kPa *versus* WT 25 kPa and polystyrene with bar color as in Figure 2, ^###^*p* < 0.001 WT 25 kPa and polystyrene ± SD208 (ANOVA/Student-Newman-Keuls). The scale bar represents 100 μm, 10× original magnification. *Green* = alpha smooth muscle actin, *red* = phalloidin, *yellow* = merged. *C,* total TGF-β levels were significantly decreased with TGF-β immunodepletion or neutralization. Isotype control: *blue bar*, anti-TGF-β: *brown bar*. Results shown as mean ± SD from three independent experiments (shown in individual data points) performed in technical duplicates. ∗∗∗*p* < 0.001 anti-TGF-β *versus* isotype control (ANOVA/Student-Newman-Keuls). *D,* TGF-β neutralization or immunodepletion decreased the ability of WT BMDM CM to induce myofibroblast differentiation in MLF by immunofluorescence as quantified in *E,* unconditioned media was set to 100%. Unconditioned media: *beige* bar, CM-no depletion: *green bar*, CM- 20 μg control Ab: *blue* bar; CM- 20 μg TGF-β Ab: *brown bar*. Results shown as mean ± SD from three independent experiments (shown in individual data points) performed in technical duplicates. ∗*p* < 0.05 control *versus* TGF-β immunodepletion, ∗∗∗*p* < 0.001 control *versus* TGF-β neutralization (ANOVA/Student-Newman-Keuls). The scale bar represents100 mm, 20× original magnification, colors as in A. BMDM, bone marrow derived macrophage; CM, conditioned media; MLF, mouse lung fibroblast.

As macrophages can produce TGF-β (20, 22, 23, 24, 25, 26, 27), we next tested if TGF-β from macrophages induces myofibroblast differentiation, using complementary techniques. The effect of the TGF-β receptor kinase inhibitor, SD208, on myofibroblast differentiation was measured upon incubation of fibroblasts with CM from WT BMDM that were plated on various stiffnesses. The stiffness-mediated profibrotic effect of macrophage CM as described above, is abrogated upon treatment of the fibroblasts with SD208 (Figs. 3, *A* and *B* and S6*B*). As TGF-β initiates complex canonical and non-canonical intracellular signals (28, 29), we examined the role of the canonical TGF-β pathway by downregulating SMAD proteins. SMAD3 siRNA downregulated SMAD3 protein (by approximately 71–76%; Fig. S2, *A* and *B*) and blocked the capacity of WT BMDM CM to induce myofibroblast differentiation in fibroblasts, compared to non-targeting siRNA (Fig. S2, *A*–*D*). These data demonstrate that WT BMDM conditioned media initiates canonical TGF-β intracellular signals. In order to definitively demonstrate that TGF-β is the predominant TRPV4 and stiffness-dependent pro-fibrotic factor, the effect of either neutralization or immunodepletion of WT BMDM conditioned media on myofibroblast differentiation was determined. Neutralization or immunodepletion of TGF-β using an affinity purified polyclonal antibody reduced the TGF-β in the WT BMDM CM by ∼80% and decreased myofibroblast differentiation by 50 to 100% compared with that from isotype control antibody (Figs. 3, *C*–*E* and S6*C*). Taken together, these data strongly indicate that TGF-β from macrophages initiated canonical TGF-β signaling in a TRPV4 and matrix-stiffness dependent manner, that drives myofibroblast differentiation of fibroblasts.

### *Trpv4* KO macrophages have impaired TGF-β activation

**Figure 4:**
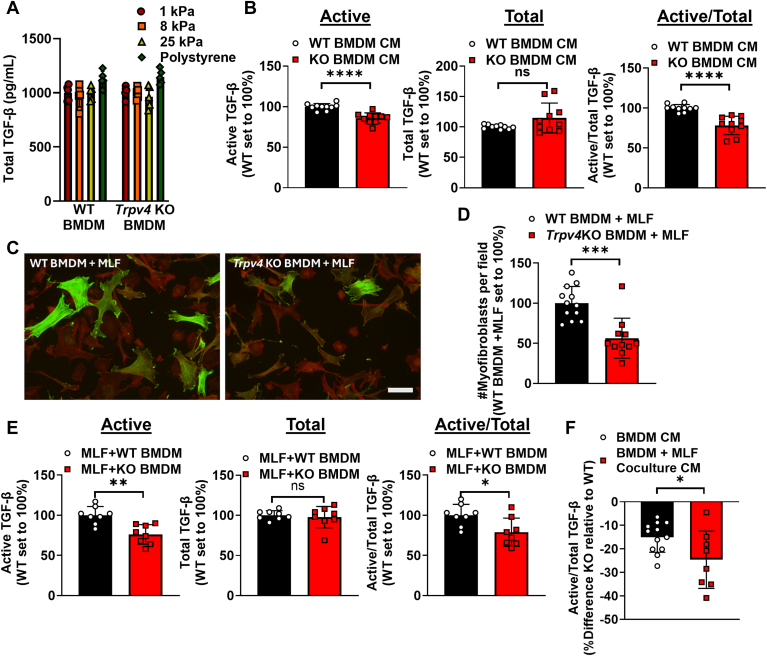
**TRPV4 in macrophages is required for optimal TGF-β activation either in monolayers or coculture with fibroblasts.***A,* total TGF-β was measured from CM from WT and *Trpv4* KO BMDMs plated on pathophysiologic range matrix stiffnesses by ELISA. Results shown as mean ± SD from five independent experiments (individual points shown) in technical duplicates. *B,* active TGF-β was measured upon transfer of CM from WT and KO BMDMs to MLECs by luminescence, total TGF-β was measured by ELISA. Results shown as mean ± SD from five independent experiments in technical duplicates (individual points shown). ∗∗∗∗*p* < 0.0001 WT BMDM CM *versus* KO BMDM CM (unpaired 2-tailed *t* test). *C,* BMDMs from WT and *Trpv4* KO were mixed with WT MLFs and cocultured together for 48 h. Myofibroblasts per field (n = 3 independent experiments, 12 and 11 total fields for WT and *Trpv4* KO, respectively) were determined by immunofluorescence as quantified in *D,* results shown as mean ± SD from three independent experiments in at least technical triplicates (individual points shown). ∗∗∗*p* < 0.001WT *versus Trpv4* KO BMDMs (unpaired 2-tailed *t* test). The scale bar represents 100 μm, 10× original magnification. *Green* = alpha smooth muscle actin, *Red* = phalloidin. *E,* active and total TGF-β were measured by transferring CM from WT MLF + WT or *Trpv4* KO BMDM coculture on PS to MLEC or ELISA, respectively. Results shown as mean ± SD from four independent experiments in technical duplicates (individual points shown). ∗∗∗∗*p* < 0.0001 WT BMDM + MLF *versus* KO BMDM + MLF (unpaired 2-tailed *t* test). *F,* percent difference in *Trpv4* KO relative to WT for CM from BMDM alone or in coculture with WT fibroblasts. Results shown as mean ± SD from at least four independent experiments in technical duplicates (individual points shown). ∗*p* < 0.05 BMDM CM *versus* coculture CM (unpaired 2-tailed *t* test). BMDM, bone marrow derived macrophage; CM, conditioned media; MLEC, mink lung epithelial TGF-β reporter cell; MLF, mouse lung fibroblast.

As data reveals that TGF-β is the predominant soluble factor secreted by macrophages that drives myofibroblast differentiation, we determined the antigenic level of TGF-β in the WT and *Trpv4* KO BMDM CM. Surprisingly, total antigenic TGF-β levels were similar and independent of substrate stiffness in the WT and *Trpv4* KO BMDMs (Fig. 4*A*). In order to resolve the apparent divergent results of immunodepletion/Smad3 signaling findings with that of equal TGF-β abundance (Fig. 3, *D* and *E*), we determined the level of TGF-β activation using a mink lung epithelial TGF-β reporter cells (MLEC) as described (30, 31). WT BMDM CM has more active TGF-β, and the active/total TGF-β ratio was significantly higher in WT *versus Trpv4* KO BMDM CM (Fig. 4*B*).

As cell-cell contact is known to robustly activate TGF-β through cellular force mechanisms (31, 32), we compared the TGF-β activation level and myofibroblast differentiating effect using macrophage-fibroblast coculture systems. Coculture with *Trpv4* KO BMDM decreased the number of myofibroblasts by approximately 42% as compared to WT BMDM (Figs. 4, *C* and *D* and S6*D*), further supporting the conclusion that TRPV4 in BMDMs drives myofibroblast differentiation that is more pronounced upon coculture than media transfer experiments as in Figure 2. The extent of the difference in the TRPV4-dependent TGF-β activation capacity of monolayers was approximately half of that of the coculture system, supporting previous work of the dependency of cell-cell contact on TGF-β activation (Figs. 4, *E* and *F* and S3).

### Activation of TGF-β by TRPV4 depends on actinomyosin cytoskeleton function

**Figure 5:**
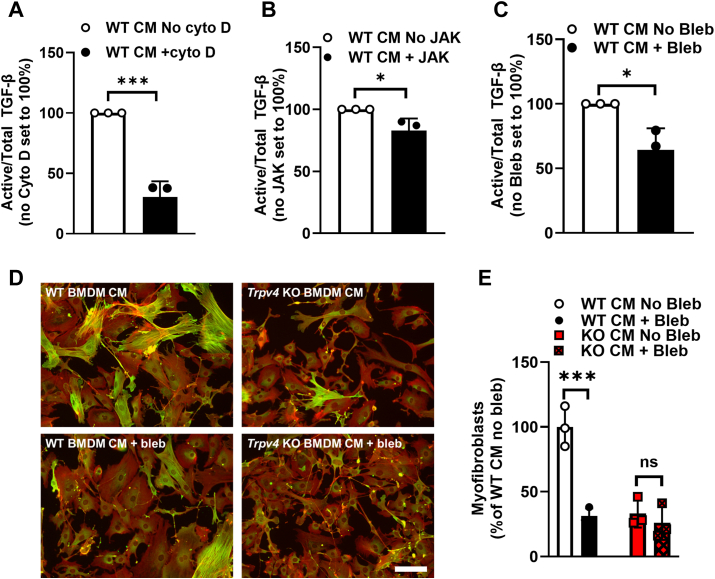
**TRPV4 mediates actinomyosin-dependent TGF-β activation in macrophages to drive myofibroblast differentiation.** CM from differentiated WT and *Trpv4* KO BMDMs were treated ± *A*, cytochalasin D (cyto D; 5uM), *B*, Jaksplakinolide (JAK; 0.1 μM), or *C*, blebbistatin (Bleb, 10 μM). Active TGF-β was measured upon transfer of CM to MLEC. Results shown as mean ± SD from three independent experiments (shown in individual data points) in technical duplicates. ∗∗∗*p* < 0.001 No cyto D *versus* + cyto D ∗*p* < 0.05 WT no Bleb or no JAK *versu*s + Bleb or JAK (unpaired 2-tailed *t* test). *D,* myofibroblast differentiation in WT MLFs treated with WT BMDM CM ± bleb was read out by immunofluorescence as quantified in *E,* results shown as mean ± SD from three independent experiments (shown in individual data points) in technical duplicates. ∗∗∗*p* < 0.001 +WT bleb *versus* WT no bleb (ANOVA, Tukey’s multiple comparisons). The scale bar represents 100 μm, 10× original magnification. *Green* = alpha smooth muscle actin, *red* = phalloidin, *yellow* = merged. BMDM, bone marrow derived macrophage; CM, conditioned media; MLF, mouse lung fibroblast.

We previously published that TRPV4 activity potentiates TGF-β1–induced actomyosin remodeling in fibroblasts (9). To that end, we determined the effect of inhibiting actinomyosin stability and function on macrophage activation of TGF-β and the myofibroblast differentiating capacity of the macrophage conditioned media. Cytochalasin D (destabilizes actin) and jaksplakinolide (blocks actin turnover) decreased the activation of TGF-β (Fig. 5, *A* and *B*) without a change in total TGF-β (Fig. S4, *A*–*D*) (33, 34). As myosin binds actin fibers to generate force (35), we tested the effect of blebbistatin, a myosin II ATP-ase inhibitor, on TGF-β activating capacity of WT BMDM. Similarly, blebbistatin reduced the TGF-β activating capacity without affecting its secretion (Figs. 5*C* and S4, *E* and *F*). Importantly, blebbistatin treatment of WT BMDMs reduced their capacity to induce myofibroblast differentiation to the level of *Trpv4* KO BMDMs while having no effect on the myofibroblast differentiation capacity of the *Trpv4* KO BMDMs (Figs. 5, *D* and *E* and S6*E*). These latter findings suggest that the entirety of the TRPV4 effect is a consequence of its capacity to regulate cytoskeletal stability. Together, these data implicate the critical role for the actinomyosin cytoskeleton in responding to TRPV4 initiated signals to activate TGF-β and drive myofibroblast differentiation.

### Activation of TGF-β is dependent on the C-terminal domain, actinomyosin binding region on TRPV4

**Figure 6:**
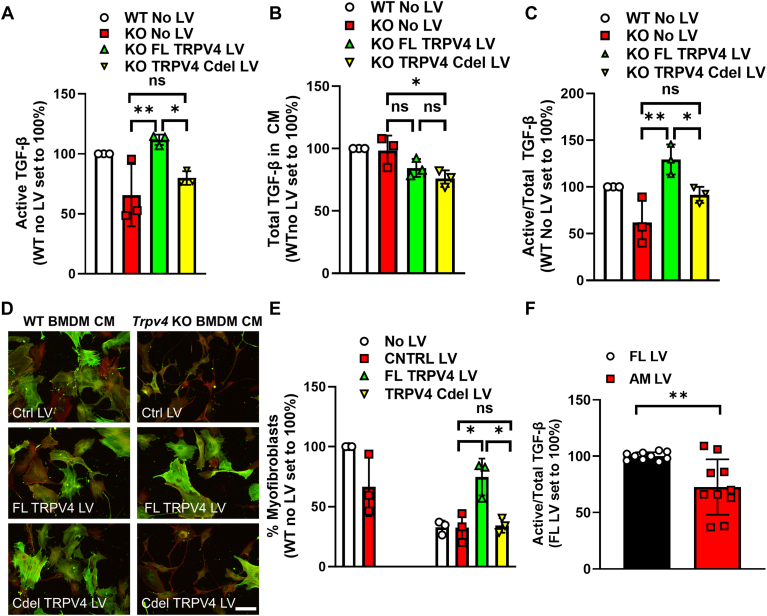
**Deletion of TRPV4’s intracellular C-terminal domain (site of actin and myosin binding) inhibits TGF-β activation and myofibroblast differentiation.** CM from differentiated WT and *Trpv4* KO BMDMs were treated with myc-tagged EV, FL *TRPV4*, or C-terminal deleted (Cdel) *TRPV4* lentiviral vectors. *A,* active TGF-β was measured upon transfer of CM from WT and KO BMDMs ± LV constructs to MLEC by luminescence (WT No LV: white bar, KO No LV: *red* bar, KO FL TRPV4 LV: *green*, KO TRPV4 Cdel LV: *yellow*). Results shown as mean ± SD from three independent experiments (individual points shown) in technical duplicates. ∗∗*p* < 0.01 KO FL TRPV4 LV *versus* KO no LV, ∗*p* < 0.05 KO FL TRPV4 LV *versus* KO Cdel LV (ANOVA/Sidak’s multiple comparisons). *B,* total TGF-β on CM was measured by ELISA. Results shown as mean ± SD from three independent experiments (individual points shown) in technical duplicates. ∗*p* < 0.05 KO no LV *versus* KO TRPV4 Cdel LV (bar color per A, ANOVA/Sidak’s multiple comparisons). *C,* values obtained from *A* and *B* were used to calculate Active/Total TGF-β. Results shown as mean ± SD from three independent experiments (individual points shown) in technical duplicates. ∗∗*p* < 0.01 KO no LV *versus* FL TRPV4 LV, ∗*p* < 0.05 KO FL TRPV4 LV *versus* KO Cdel LV (bar color per A, ANOVA/Sidak’s multiple comparisons). *D,* CM from WT and *Trpv4* KO BMDMs treated with EV, FL *TRPV4* or Cdel *TRPV4* was added to WT MLFs and myofibroblast differentiation was read out by immunofluorescence as quantified in *E,* results shown as mean ± SD from three independent experiments (individual points shown) in technical duplicates. ∗*p* < 0.05 control (CNTRL) LV *versus* FL TRPV4 LV or KO Cdel LV (No LV: white bar, CNTRL LV: *red* bar, FL TRPV4 LV: *green bar*, TRPV4 Cdel LV: *yellow* bar (ANOVA/Sidak’s multiple comparisons). The scale bar represents 100 μm, 20× original magnification. *Green* = alpha smooth muscle actin, *Red* = phalloidin, *Yellow* = merged. *F,* CM from *Trpv4* KO BMDM treated with FL TRPV4 or TRPV4 actin binding site mutant (AM) was analyzed for active/total TGF-β. Results shown as mean ± SD from five independent experiments in technical duplicates (individual points shown) ∗∗*p* < 0.01 FL TRPV4 LV *versus* TRPV4 actin mutant (AM) LV (unpaired 2-tailed *t* test). BMDM, bone marrow derived macrophage; CM, conditioned media; MLEC, mink lung epithelial TGF-β reporter cell; MLF, mouse lung fibroblast.

Given our finding of the importance of the actinomyosin cytoskeletal function and known binding of the C-terminal intracellular tail to actinomyosin (36), we determined the effect of deletion/mutation of the TRPV4 actinomyosin binding domain/site (C-terminal domain deletion AA 723–871) on its capacity to activate TGF-β and drive myofibroblast differentiation. Lentiviral mediated expression of a TRPV4 mutant lacking its C-terminal domain resulted in a suppression of TGF-β activation and myofibroblast differentiation to the level of untransduced or control vector-transduced *Trpv4* KO BMDMs (Fig. 6, *A*–*E* and S6*F*). The transfection efficiency of the FL and Cdel TRPV4 LV were relatively equal, as measured by immunoblot (Fig. S5).

To further substantiate the importance of actinomyosin binding to the TRPV4 C-terminal domain, we tested the effect of expression of a TRPV4 actinomyosin binding site specific scrambled mutant (AA 746–779) on the macrophages’ capacity to activate TGF-β (37). Similarly, expression of the actinomyosin specific mutant (AM LV) in the *Trpv4* KO BMDMs demonstrated an impaired capacity (∼30%) to activate TGF-β compared to that of FL TRPV4 (Fig. 6*F*), despite relatively equal transduction/expression efficiency (Fig. S5). These data provide further evidence for the importance of TRPV4-actinomyosin binding site in mediating TGF-β activation and myofibroblast differentiation. Taking collectively with the presented *in vitro* and *in vivo* data, we convincingly demonstrate that TRPV4’s actinomyosin binding function drives TGF-β activation, myofibroblast differentiation, and experimental pulmonary fibrosis.

## Discussion

**Figure 7:**
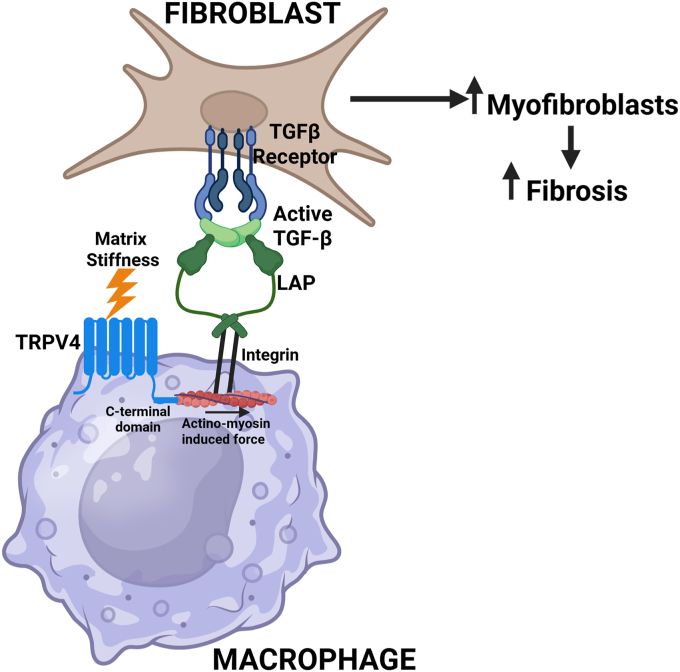
**Working model of TRPV4-dependent TGF-β activation upon macrophage-fibroblast interaction.** Upon macrophage sensing of matrix stiffness, TRPV4 C-terminal domain binds to actinomyosin, thereby generating force on the integrin. Based on extensive literature the integrin binds to the latency associated peptide within the latent TGF-β complex, thereby exposing the cryptic TGF-β receptor binding site. The active TGF-β binds to the TGF-β receptor on the fibroblast to enhance collagen production, myofibroblast differentiation and ultimately pulmonary fibrosis.

Our foundational work shows that the mechanosensitive, cation ion channel, TRPV4, plays a key role in myofibroblast differentiation, *in vivo* pulmonary fibrosis, and the macrophage response to infection (8, 9, 11, 12). The current work seeks to determine if TRPV4 in macrophages mediates the pro-fibrotic pathway through paracrine signaling to fibroblasts and if so through what molecular mechanism. Collectively, the data presented here show that macrophage TRPV4 is required to secrete a pro-fibrotic factor that leads to myofibroblast differentiation and collagen-1 production (Fig. 7). As TGF-β is a cytokine or growth factor that is pro-fibrotic, our data supports that the TRPV4-dependent pro-fibrotic factor produced by the macrophage is TGF-β. Although TRPV4 didn’t play a role in secretion of TGF-β as levels were equal in WT and *Trpv4* KO macrophages, TRPV4 is required for the optimal activation of TGF-β. Furthermore, activation of TGF-β by macrophages requires TRPV4’s C-terminal actinomyosin binding domain along with an intact and functional actinomyosin cytoskeleton. Taken together, this work demonstrates the novel TRPV4-TGF-β axis whereby the C-terminal actinomyosin binding domain of TRPV4 in macrophages is required for force-dependent activation of TGF-β resulting in myofibroblast differentiation and experimental pulmonary fibrosis.

The etiology of fILDs, especially those related to connective tissue diseases, has been shown to be driven in part by immune mechanisms (1). Yet more recently, even the idiopathic form, idiopathic pulmonary fibrosis (IPF), has also been shown to involve both the innate (macrophages, neutrophils) and adaptive (T cells) immune system (16, 17). Specifically, several lines of evidence support that macrophages play a role in fibrogenesis such as *i)* unique subsets of pro-fibrotic macrophages are found on single-cell RNA sequencing of fibrotic human and mouse lung tissue, *ii)* macrophages are a primary source of active TGF-β, and *iii)* macrophage depletion protects against fibrosis, but the mechanism of the macrophage induction of fibrosis is not clearly defined (17, 20, 22, 23, 24, 25, 26, 27, 38). IPF pathogenesis is known to involve, among other processes, epithelial injury, immune cell infiltration, and fibroblast-to-myofibroblast transition driving extracellular matrix deposition and pro-fibrotic cytokine production (*e.g.*, TGF-β) (32, 39, 40, 41, 42). This leads to tissue stiffening, and recruitment of monocyte-derived macrophages (MDM), thereby establishing a self-perpetuating cycle of fibrotic foci formation, a hallmark of IPF (20, 22, 43, 44, 45, 46).

Single cell RNA sequencing of animal and human bronchoalveolar lavage, and samples from fibrotic lung tissue reveal distinct monocyte-derived macrophages and alveolar macrophage populations with pro-fibrotic features (24, 25, 26, 27). Specifically, TRPV4 is highly upregulated in macrophages from IPF patients as shown in single cell RNA sequencing data from the human IPF lung atlas (47). Lung macrophage phenotype varies based on their ontogeny, location in the lung, and their surrounding microenvironment (48, 49). However, how the lung microenvironment affects the phenotype of macrophages has been less explored. The lung microenvironment changes during the aging process, which leads to many chronic lung diseases, such as IPF and senescence-associated secretory phenotype (50, 51). In fact, aging leads to changes in the matrix biophysical properties and components, and in quantity and function of macrophage populations, similar to that seen in IPF (52, 53, 54). Emerging data suggests that the lung macrophage phenotype is plastic and comprises a pro-fibrotic “M2-like” population is responsible for TGF-β production and secretion (22). Our data demonstrates that TRPV4 shifts the macrophage population towards a pro-fibrotic, TGF-β activating phenotype in response to matrix mechanical stiffness in the pathophysiologic range, as seen in IPF. This study only used bone marrow-derived macrophages differentiated with M-CSF (50 ng/ml, 7 days). Future work will determine the pro-fibrotic effect of TRPV4 in the alveolar *versus* interstitial macrophage given they interact with different aspects of the lung microenvironment. Parenthetically, in other work we have published there was no difference in the TRPV4 dependence of the LPS response in BMDMs and alveolar macrophages from mice and humans (11, 12).

TGF-β is a unique cytokine as it is secreted in its inactive form and can be activated, in part, through several mechanical force dependent mechanisms (55). The inactive or latent form of TGF-β is in a trimeric complex with the latent TGF-β binding proteins and latent TGF-β prodomain (LAP) (56). latent TGF-β binding proteins are known to bind fibronectin and fibrillin, which sequesters the TGF-β-LAP complex into the extracellular matrix, thereby localizing TGF-β activity (57, 58, 59). In turn, integrins in the β1 (αvβ3 and αvβ6) and β2 (like αMβ2 or Mac-1) family can bind the Arg-Gly-Asp (RGD) integrin-specific binding motif in the LAP (18, 28). Cell-cell or cell-matrix force can facilitate conformational change of the TGF-β-LAP complex exposing active TGF-β to its cognate receptor in order to initiate intracellular signaling (59, 60, 61). Our work supports cytoskeletal binding to TRPV4’s C-terminal actinomyosin domain, which is the initiating event that is suggested to result in force generation leading to TGF-β activation by monocyte-derived macrophages. TRPV4 can control cytoskeletal-induced force through several possible mechanisms. TRPV4 may affect integrin function directly by facilitating integrin binding to the LAP RGD domain, as TRPV4 is needed for the β1 integrin functions of cell matrix adhesion, cytoskeletal organization and integrin to integrin signaling in other cell types (62, 63). Alternatively, the TRPV4 dependency on Glycoprotein A repetitions predominant unction, TGF-β-LAP activating and signaling receptor, on immune cells such as macrophages and T cells, has yet to be explored (64, 65). The literature supports that cells in contact with each other activate more TGF-β than those that are not in contact (60). As our data shows that TRPV4 binds to the cytoskeleton *via* its C-terminal tail to generate force, we hypothesize that the *Trpv4* KO BMDMs would not generate as much force on cell-cell contact, lessening the macrophage’s capacity to expose the cryptic TGF-β receptor binding site of the latent TGF-β complex (29, 60). This would result in an impaired ability of the latent TGF-β complex to bind to its nascent receptor as evident by the impaired measured TGF-β activation. Other possibilities include potential differences in integrin binding or in other cell adhesion proteins such as connexin or cadherins between the WT and *Trpv4* KO BMDMs (31, 60, 61).

Interestingly, our data shows that loss of TRPV4 induces a small change in TGF-β activation as compared to the large protection from both myofibroblast differentiation and experimental pulmonary fibrosis (Figure 1, Figure 2 and 4). One potential explanation is that the myofibroblast differentiation response to TGF-β in not linear and is quite variable in different cells and systems (66, 67, 68). For example, if the cell is stimulated with a small amount of active TGF-β but over a prolonged period of time, there may be a longer lasting pro-fibrotic effect (67, 68). The consequences of linearity and exposure time are not considered in our *in vitro* experimental system. Nonetheless, we saw a clear *in vivo* effect that mimicked our *in vitro* findings. There is a possibility that this *in vivo* effect may also be due to TRPV4 deletion in a portion type II alveolar epithelial cells that may be affected by the LysMCre promoter (69). Both murine and human epithelial cells have been shown to activate TGF-β (60). However, we show, using multiple techniques, that CM from WT BMDMs alone can induce myofibroblast differentiation and collagen-1 production (Fig. 2) due to active TGF-β (Fig. 4), and these functions are significantly decreased with TRPV4 deletion (Figs. 2 and 4). Although we saw abrogation of the myofibroblast differentiation effect with neutralizing TGF-β, it remains possible that there is an additional TRPV4-dependent macrophage factor that is synergistic with TGF-β′s pro-fibrotic actions, such as platelet-derived growth factor and Wnt signaling (66, 68, 70, 71). Other putative TRPV4 and stiffness-dependent pro-fibrotic factors will be investigated in the future.

Taken together, our work advances the understanding of the mechanism of TGF-β activation by macrophages that lead to myofibroblast differentiation and pulmonary fibrosis. A strength of our observations is that complementary gain and loss of function and molecular antibody based, and overexpression systems reveal similar results. However, there are some limitations to the methodology and interpretations of our study. All of our data is using mouse *in vitro* and *in vivo* systems that may exhibit different biologic response than humans. However, this is somewhat mitigated by the fact that IPF macrophages exhibit high levels of TRPV4 and TGF-β. Although we have solid evidence of the existence of a macrophage-TRPV4-cytoskeleton-TGF-β activating axis, other previously described mechanisms exist for activation of TGF-β (18). Even though calmodulin, a cytoskeleton activating protein, is known to bind to the intracellular C-terminal domain of TRPV4, its site of binding is distinct from that of the actinomyosin binding domain (72). Furthermore, the level of impairment of TGF-β activation among the TRPV4 actinomyosin scrambled mutant and C-terminal deleted mutant is similar. These data suggest that the calcium-dependent calmodulin binding to TRPV4 is not a significant contributor to the overall results. Calcium signaling has been shown to be indirectly involved in TGF-β activation (73). However, it is experimentally difficult to discern if either TRPV4-dependent calcium function or other macrophage calcium channels play a major role in our findings, due to the protean effects of intracellular calcium on general macrophage function.

In conclusion, fibrosis is a consequence of multiple coordinated biological processes. Pulmonary fibrosis requires both a mechanical and soluble signal. We have previously shown that TRPV4 is essential for *in vivo* pulmonary fibrosis and the macrophage response to infection using global *Trpv4* KO mice. The data presented herein demonstrates that macrophage TRPV4 is a key effector in the fibrosis process by optimally activating TGF-β, thereby driving myofibroblast differentiation and experimental pulmonary fibrosis. The TRPV4 function is dependent on the presence of the actinomyosin binding domain on its C-terminal intracellular tail and on an intact and functioning cytoskeleton. Targeting TGF-β directly has been unsuccessful likely given its pleotropic effects (19). We speculate that focusing on localized macrophage TRPV4-dependent activation of TGF-β may elicit a more favorable response and therefore may serve as a druggable target in the treatment of pulmonary fibrosis.

## Experimental procedures

### Sex as a biological variable

Both male and female BMDMs and transgenic mice were used in this study.

### Antibodies and reagents

Blebbistatin, jaksplakinolide, cytochalastin D, ALK5 inhibitor (SD208), and antibodies to alpha smooth muscle actin and collagen-1 were from Sigma Aldrich. Alexa Fluor 594-phalloidin and Alexa Fluor-conjugated secondary antibodies were from Invitrogen. Antibody to GAPDH was obtained from Fitzgerald; glass-bottom or plastic plates containing activated polyacrylamide gels of 1, 8, or 25 kPa were custom made by Matrigen Life Technologies. MLEC were a gift from Dr Raed Dweik. The TGF-β ELISA was purchased from R&D systems, as was the TGF-β antibody used for pull-down and neutralization. Smad and TRPV4 antibodies were purchased from Cell Signaling, and the siRNA was from Dharmacon. The Isotype control antibody for pull-down and neutralization experiments was from Biolegend. TRPV4 full-length, C-terminal deleted, and actinomyosin mutant lentivirus was purchased from VectorBuilder. In order to generate an actomyosin binding-deficient construct we mutated the nucleic acid sequence of TRPV4 corresponding to amino acids 746 to 779 (RSFPVFLRKAFRSGEMVTVGKSSDGTPDRRWCFR). We mutated these 34 amino acids to a 17 X GS repeat in order to maintain proper structure of the carboxy-terminal tail of TRPV4 and conserve all other functions. C57BL/6 mice were from The Jackson Laboratory. *Trpv4* KO mice were a gift from David Zhang.

### Bleomycin-induced pulmonary fibrosis model

Induction of pulmonary fibrosis was performed in a single dose bleomycin model in *Trpv4*^fl/fl^ and *Trpv4*^LysMCre^ mice generated by us. Bleomycin (1.5U/kg) or phosphate-buffered saline (as a control) was instilled intratracheally as previously published (9). Animals were euthanized at 14 days after bleomycin and left lung collected for hydroxyproline (a marker of collagen deposition) and collagen-1 by immunoblot (9). Right lungs were inflated with OCT as previously published (9). Static compliance, elastance, and resistance measurements were performed on the FlexiVent animal ventilator (Scireq). Anesthetized, tracheostomized, paralyzed, and mechanically ventilated mice were used to perform P-V loop measurements, to obtain lung compliance. Bronchoalveolar lavage was performed to determine total cell counts and differentials and total protein as a marker of vascular leak as described previously (9, 11). All animal protocols were performed according to guidelines approved by the Cleveland Clinic Institutional Animal Care and Use Committee (IACUC).

### Cell culture, conditioned media transfer, and immunoblotting

Primary MLFs were derived from 7- to 10-week-old WT mice and propagated in complete media (MEM supplemented with 10% fetal bovine serum (FBS)) as previously described (9, 74). MLFs were obtained by outgrowth of fragments of collagenase digested lung tissue as we published (9). MLF media was changed every 2 to 3 days and MLF were passaged with trypsin-EDTA when the cells reached 80 to 90% confluence, for up to five passages (74). WT MLFs were cultured the same way on tissue culture plastic (without polyacrylamide gels) and pretreated ± ALK5 inhibitor (SD208) or SMAD2/SMAD3 siRNA for experiments with WT CM added to fibroblasts, or in coculture with BMDMs. Primary BMDMs were harvested from 8–12-week-old WT or *Trpv4* KO mice and maintained in 10% FBS/RPMI. BMDMs were differentiated in recombinant mouse macrophage colony stimulating factor (50 ng/ml, Peptrotech) as previously published (11, 12). BMDMs were plated in 10% FBS/RPMI on tissue culture plastic or on plastic plates containing polyacrylamide gels of 1, 8, or 25 kPa. Then, the BMDMs were pretreated ± blebbistatin, jaksplakinolide, or cytochalasin D for 24h, then the conditioned media was removed and analyzed for total TGF-β (ELISA), active TGF-β (MLEC – see below), or for its ability to induce myofibroblast differentiation in WT MLFs. The BMDM CM was subjected to ± TGF-β neutralization with TGF-β antibody or immunodepletion using a bead-based assay (or isotype control) (75, 76, 77), was then added to the WT MLFs (1:10 in 1% BSA/serum-free medium (SFM)-MEM) for 48 h, and myofibroblast differentiation was read out by immunofluorescence (below) or by Western blotting for collagen-1 (below).

The BMDMs were cultured on fibronectin-coated (10 μg/ml) polyacrylamide hydrogels of indicated stiffnesses. The conditioned media from these BMDMs was then transferred to fibroblasts that were plated on tissue culture plastic. For BMDM/MLF coculture assays (Fig. 4, *C*–*E*), BMDMs and MLFs were mixed prior to plating on tissue culture plastic in 10% SCM overnight in macrophage medium. The next day the BMDM/MLF cocultures were washed 2 times in SFM, and 1% BSA/SFM was added for 48 h, after which the CM was harvested and analyzed, and cocultures were stained (as below). For BMDM/MLEC coculture assays (Fig. S3), BMDMs and MLEC were mixed prior to plating on tissue culture plastic in 10% SCM overnight in macrophage culture media (RPMI 10% FBS). The next day the coculture CM was harvested and analyzed for total TGF-β by ELISA. The MLECs were washed and lysed for luminescence assays for active TGF-β, as below.

### Immunofluorescence/immunoblotting

Immunoblotting was performed as previously published. For collagen-1, GAPDH, SMAD2 and SMAD3 detection in WT MLF, cells were lysed in 1% NP-40 lysis buffer and separated on Criterion gels (Biorad) at a constant 100V and transferred to 0.45-micron PVDF membranes (Thermo Fisher Scientific) at a constant 100 mA for 2 h. For TRPV4 full length and mutant lentivirus detection in transfected BMDMS, TRPV4 antibody (LSBio) was used with the same protocol. Primary and HRP-tagged secondary antibodies were used as published and detected using an enhanced chemiluminescence system (Amersham ECL Prime Western Blotting Detection Reagent) on a UVP Biospectrum imaging system (Analytik Jena) using total time image integration as published (9, 11, 12). Band density was quantified using VisionWorks acquisition and analysis software version 8.19.17027.9424 (Analytik Jena) and normalized to GAPDH in each lane. Regarding antibody validation, the collagen-1 and GAPDH antibodies used within have been previously published by our lab (8, 9, 78), the SMAD2 and SMAD three band densities were significantly decreased with their respective siRNAs, and the TRPV4 antibody band size was specific for the mutant molecular weights.

To determine the BMDM CM effect on myofibroblast differentiation in WT MLF, WT MLF were treated as in the previous section, fixed in 4% paraformaldehyde, permeabilized with 0.5% Triton X-100, and blocked with 2% normal goat serum. To label SMA, primary SMA antibody (1:1000) was used, followed by AlexaFluor488 secondary (1:1000). F-actin stress fibers were stained by AlexaFluor 594 phalloidin (1:100). Images were acquired using a Leica DM IRB inverted microscope (Leica Microsystems) equipped with a Leica DFC 7000T camera and Leica Application Suite X (LAS X) v.3.6.0.20104 software.

WT MLF was considered myofibroblasts if the SMA and F-actin were aligned in stress fibers – and at least 30 cells/condition were counted in duplicate wells. Regarding antibody validation, the SMA, F-actin (phalloidin), and secondary antibodies used within have been previously published by our lab (8, 9, 78).

### TGF-β activity assay

Active TGF-β was determined *via* mink lung epithelial cells (MLEC), which make luciferase in response to active TGF-β *via* the plasminogen activator inhibitor-1 (PAI-1) promoter (79). BMDM CM (from 500,000 BMDM/ml) was added directly to attached MLEC for 20 h, or, for coculture experiments, MLEC and BMDM were plated directly together for 20 h. MLECs were lysed, and luminescence was determined with a luciferase assay kit (Promega) and luminometer (SpectraMax iD3, Molecular Devices, Softmax Pro Software 7.0.3).

### siRNA mediated knockdown

All siRNAs were transfected into WT MLFs using siLentFect lipid reagent (Bio-Rad) according to the manufacturer’s instructions. SMAD2-or SMAD3-specific siRNA and control scrambled siRNA duplexes were purchased from Dharmacon and used at the indicated concentrations (24 h of transfection). After transfection, cells were washed with SFM and conditioned media from WT or *Trpv4* KO BMDMs were added.

### Lentiviral constructs

Lentiviral constructs for (myc)–tagged WT TRPV4 (full length) and myc-C-terminal deleted TRPV4 (Cdel, AAs 723–871 deleted) were produced by Vector Builder. The TRPV4 actin binding site was published by Goswami *et al.* (37) For transduction, *Trpv4* KO BMDMs were exposed to 50 MOI of one of the above lentiviral constructs (or a control lentivirus) for 48 h in complete RPMI supplemented with polybrene (4 μg/ml, Santa Cruz Biotechnology) as published (80). Cells were washed three times with SFM and transferred to 1% BSA/SFM for 24 h. The BMDM conditioned media was saved and analyzed for active and total TGF-β, and the BMDMs were lysed in 1% NP-40 lysis buffer and Western-blotted for TRPV4 protein as above. Transfection efficiency was determined by Western blotting for TRPV4 and GAPDH.

### Statistical analysis

All data are expressed as means ± SD unless otherwise indicated. Statistical comparisons between control and experimental groups were performed using SigmaPlot or GraphPad Prism software. Student’s *t* test was used for two-group comparisons, whereas one-way analysis of variance (ANOVA) was used for comparisons between more than two groups. A Student-Newman-Keuls, Tukey’s test, Holm-Šídák, or Fisher’s Least Significant Difference test was used to adjust for multiple comparisons. Values of *p* ≤ 0.05 were considered statistically significant.

### Study approval

The animal studies were performed with approval by the Cleveland Clinic’s Institutional Animal Care and Use Committee (IACUC) #2624, expiration date 03/31/2027.

## Data availability

All data from this manuscript are within the main text or supplement. The Raw data will be made available upon request.

## Supporting information

This article contains supporting information.

## Conflict of interest

The authors declare that they have no conflicts of interest with the contents of this article.
