# Supplementary material for: Transient receptor potential vanilloid four in macrophages mediates TGF-β activation to drive myofibroblast differentiation and pulmonary fibrosis
Source: J Biol Chem. 2026 Jan 7;302(2):111135. doi: 10.1016/j.jbc.2026.111135 (PMC12860955; doi:10.1016/j.jbc.2026.111135)
Supplement: Supporting information [file mmc1.docx]

**Title: Transient Receptor Potential Vanilloid 4 in Macrophages Mediates TGF-β Activation to Drive Myofibroblast Differentiation and Pulmonary Fibrosis**

**Authors:** Lisa M. Grove^1^, Caitlin Snyder^1^, Adam M. Boulton^1^, Hongxia Mao^1^, Susamma Abraham^1^, Haley Brown^1^, Erica M. Orsini,^2^ Brian D. Southern,^1,2^ Mitchell A. Olman^1,2^, Rachel G. Scheraga^1,2^

Figures S1 – S6 included herein.

**Figure S 1: Bleomycin induces a downward shift of pressure volume curve in *Trpv4*^fl/fl^ mice compared to *Trpv4*^LysMCre^ mice.** Representative pressure volume loop for saline and bleomycin treated *Trpv4*^fl/fl^ (red) and *Trpv4*^LysMCre^ (blue) mice at 14 Days on FlexiVent.

**
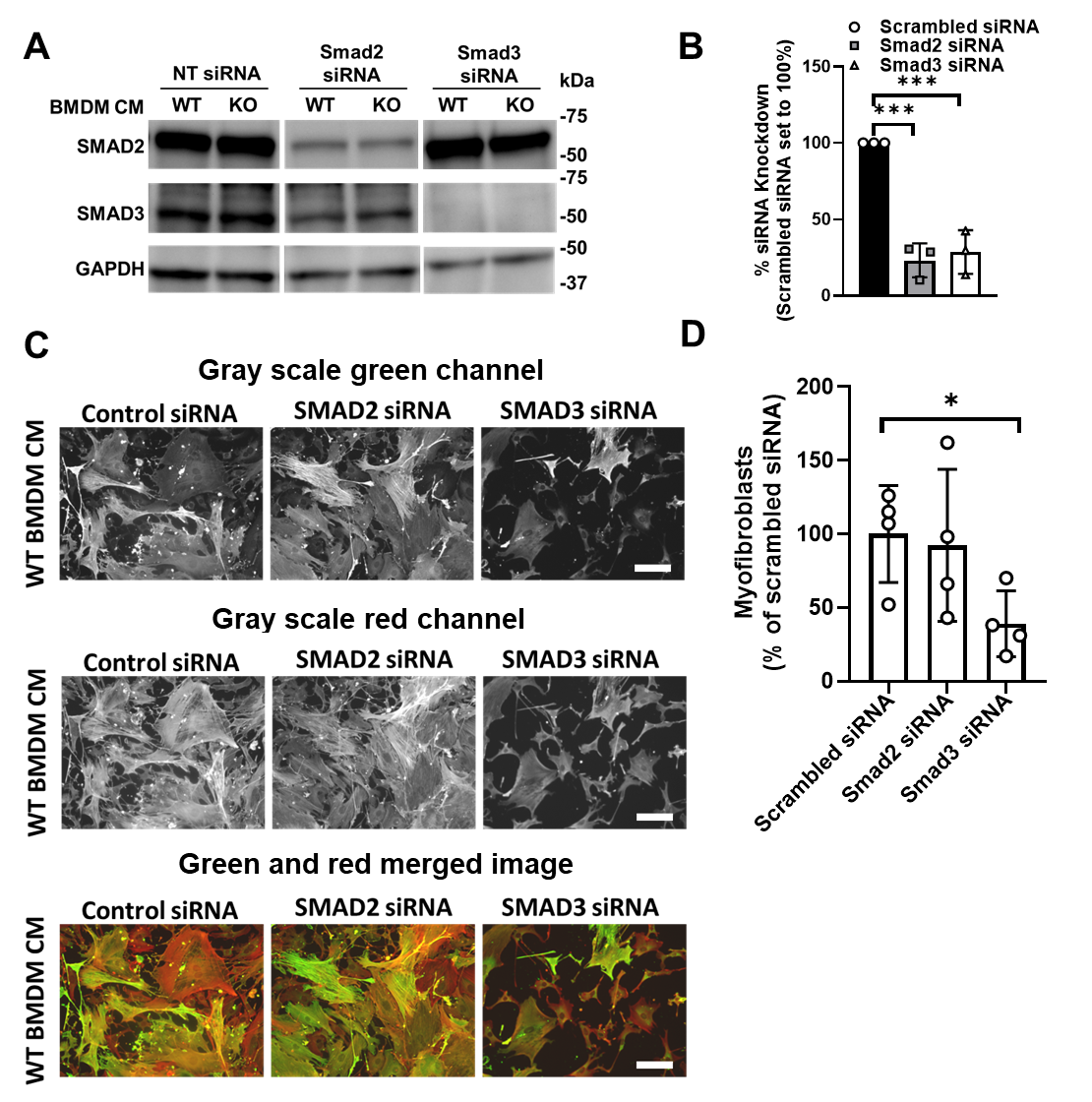
**

**Figure S 2: Macrophage induced myofibroblast differentiation is dependent on Smad3 signaling.** **A**. WT mouse lung fibroblasts (MLF) were treated with Smad 2 and Smad 3 siRNA with ~70% knockdown, as quantified in **B**. Results shown as mean ± SD from three independent experiments (individual points shown). ****p*<0.001 Scrambled siRNA vs SMAD2 or SMAD3 siRNA (ANOVA/Tukey’s multiple comparisons). **C.** Only SMAD 3 knockdown in WT MLF abrogated WT BMDM CM ability to induce myofibroblasts by immunofluorescence as quantified in **D**. Results shown as mean ± SD from four independent experiments (individual points shown) with technical duplicates. **p*<0.05 Scrambled siRNA vs Smad 3 siRNA (unpaired 2-tailed t-test). Scale bar 100µm, 10x original magnification. Green = alpha smooth muscle actin, Red = phalloidin, Yellow = merged.


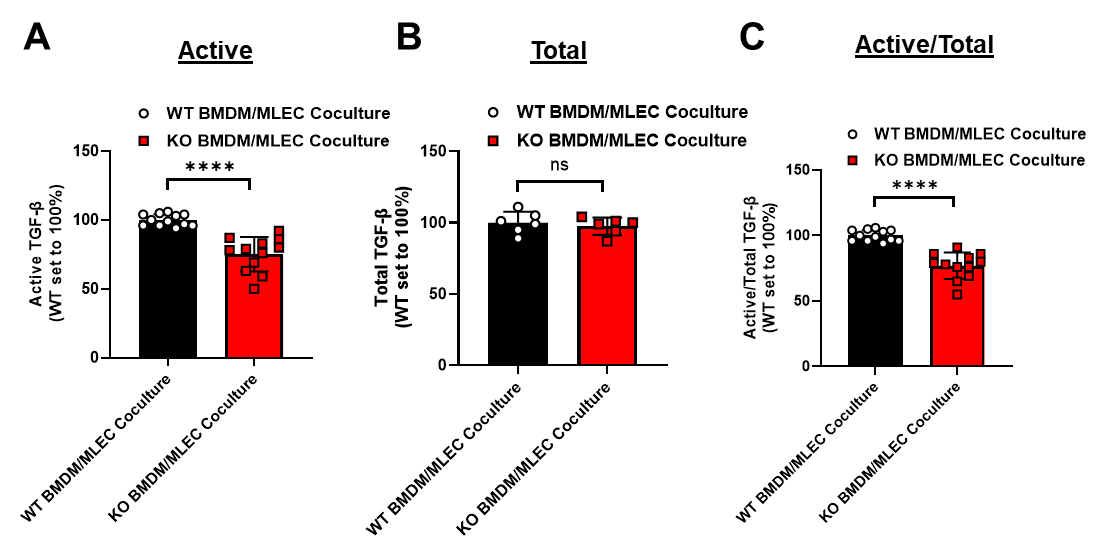


**Figure S 3: TRPV4-dependent TGF-β activation by macrophages is enhanced by interaction with MLECs. A.** Active TGF-β was measured upon WT or *Trpv4* KO BMDM coculture with MLECs. Results shown as mean ± SD from 3 independent experiments in at least technical duplicates (individual points shown) for **A**, **B**, and **C**. *****p*<0.0001 WT vs KO BMDM (unpaired 2-tailed t-test). **B.** Total TGF-β was measured using CM from BMDM and MLEC co-culture by ELISA. Not statistically significant (ns). **C.** Active/total TGF-β calculated based on A and B. *****p*<0.0001 WT vs KO BMDM (unpaired 2-tailed t-test).


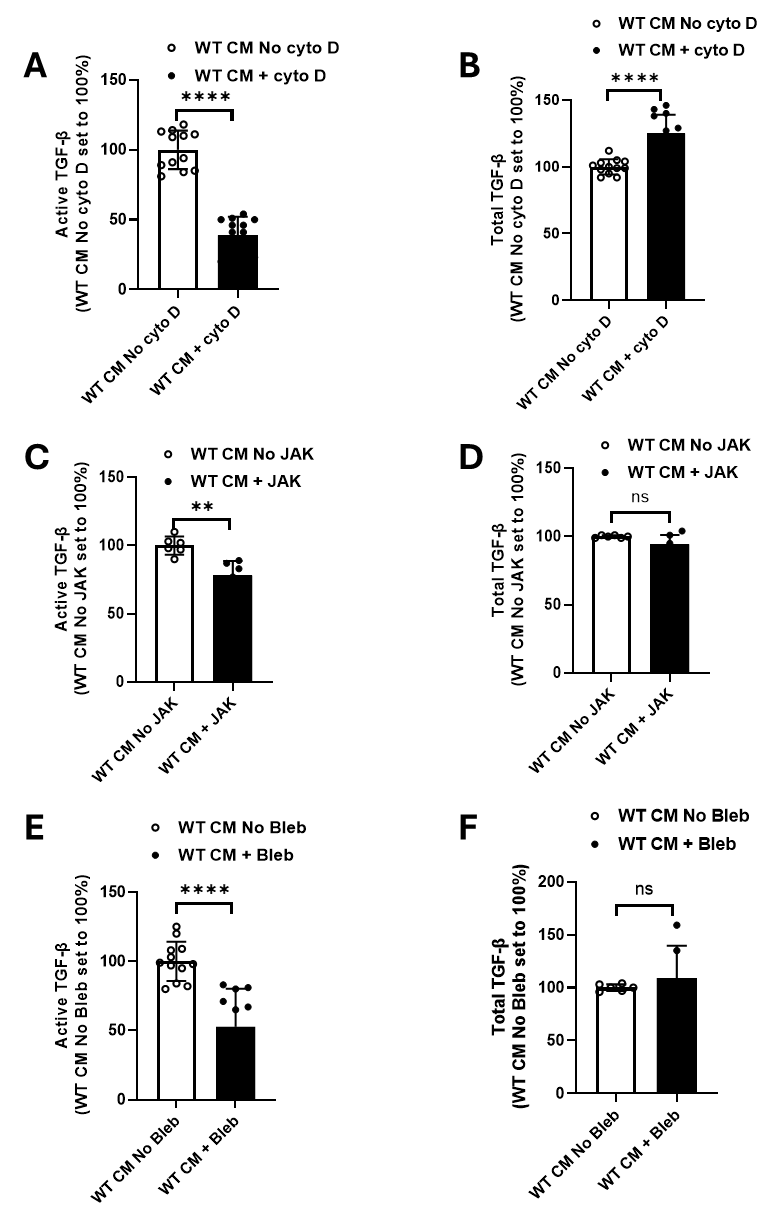


**Figure S 4: Active TGF-β is decreased with inhibition of actinomyosin without a decrease in total TGF-β.** CM from differentiated WT BMDMs were treated ± Cytochalasin D (Cyto D; 5mM) **A, B**, Jasplakinolide (JAK; 0.1mM) **C, D**, or Blebbistatin (Bleb; 10mM) **E, F** and analyzed for active TGF-β (upon transfer of CM to MLEC) or total TGF-β (by ELISA), respectively, for each inhibitor. Results shown as mean ± SD for three independent experiments with at least technical duplicates (individual points shown). *****p*<0.0001 or ***p*<0.01 no inhibitor vs inhibitor (unpaired 2-tailed t-tests).


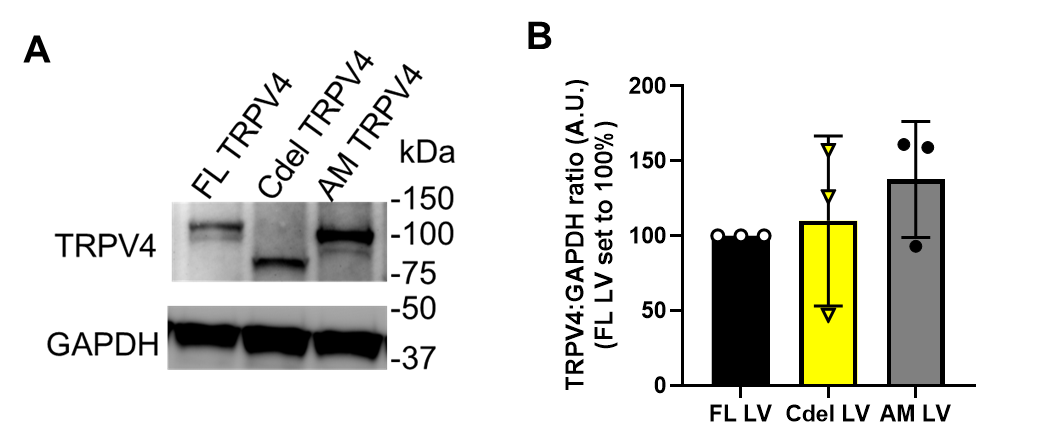


**Figure S 5: Immunoblot shows equal expression of TRPV4 lentiviral driven protein. A.** Transfection efficiency of TRPV4 full-length (FL), C-terminal deleted (Cdel), and actinomyosin scrambled (AM) mutants were evaluated by immunoblot as quantified in **B**. TRPV4 antibody binds to AA 113-133. Results shown as mean ± SD from three independent experiments (individual points shown).


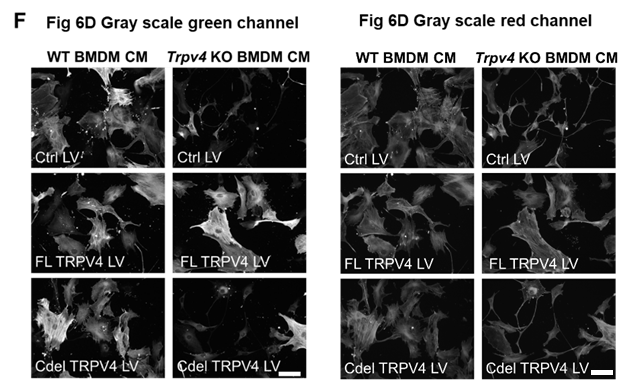

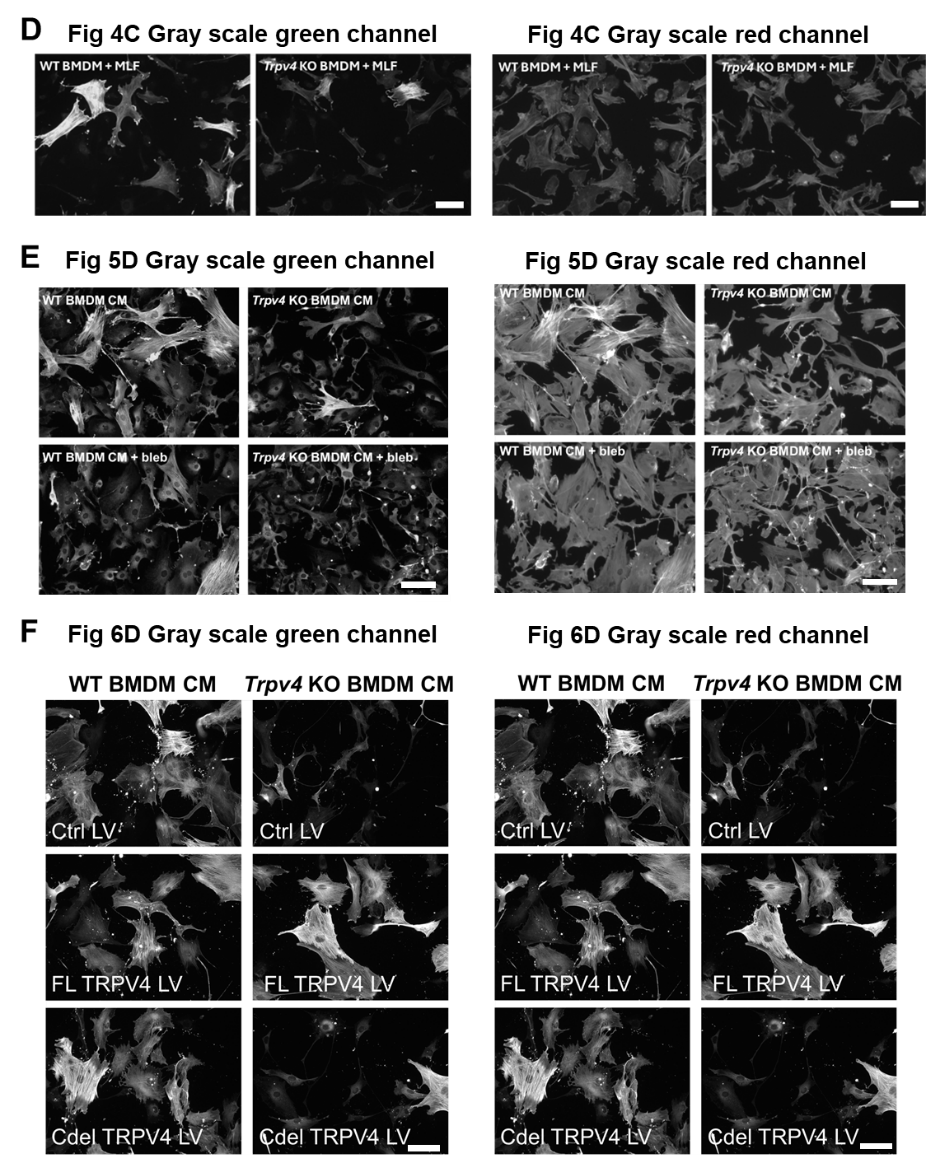

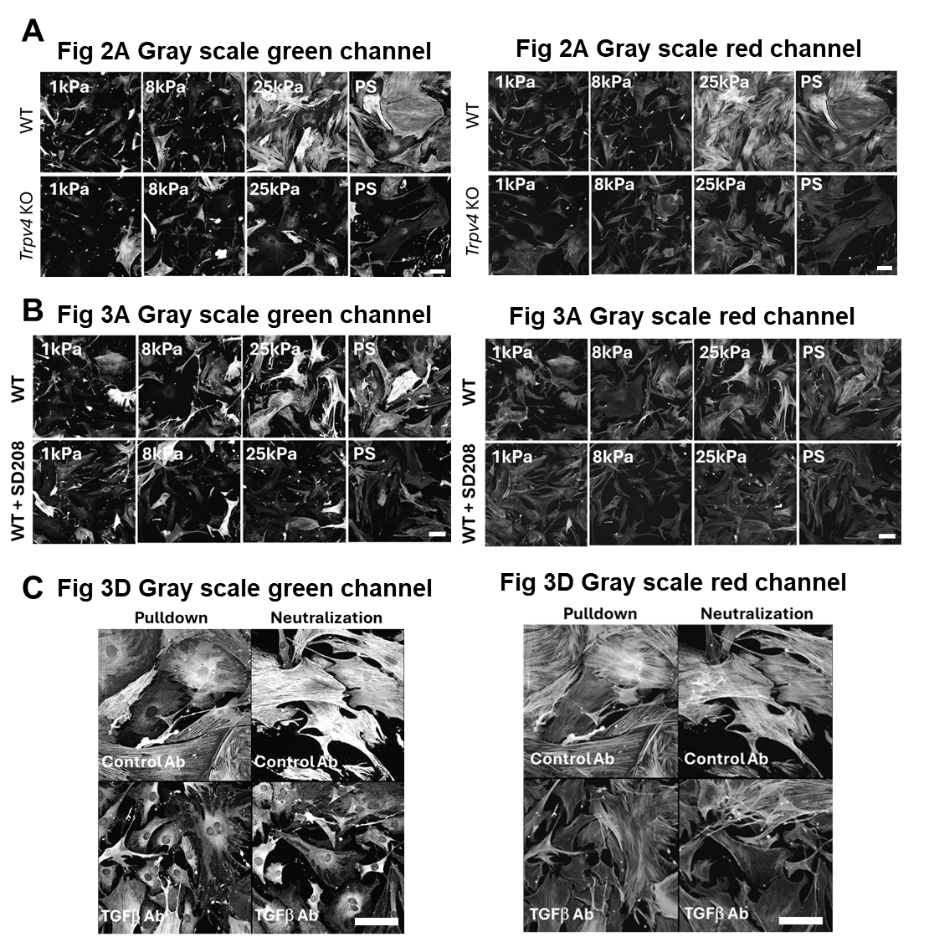


**Figure S 6: Separated gray scale images of multi-color images.** Gray scale green and red images, respectively, for multi-color images in **A**. Figure 2A and **B**. Figure 3A, both 10x original magnification, scale bars 100µm. **C.** Figure 3D, 20x original magnification, scale bars 100µm. **D.** Figure 4C, Figure 5D (**E**.), and Figure 6D (**F.**) all 10x original magnification, scale bars 100µm.
